# An External Circular Crack in an Infinite Solid under Axisymmetric Heat Flux Loading in the Framework of Fractional Thermoelasticity

**DOI:** 10.3390/e24010070

**Published:** 2021-12-30

**Authors:** Yuriy Povstenko, Tamara Kyrylych, Bożena Woźna-Szcześniak, Renata Kawa, Andrzej Yatsko

**Affiliations:** 1 Department of Mathematics and Computer Sciences, Faculty of Science and Technology, Jan Dlugosz University in Czestochowa, al. Armii Krajowej 13/15, 42-200 Czestochowa, Poland; t.kyrylych@ujd.edu.pl (T.K.); b.wozna@ujd.edu.pl (B.W.-S.); r.kawa@ujd.edu.pl (R.K.); 2 Department of Mathematics, Faculty of Civil Engineering, Environmental and Geodesic Sciences, Koszalin University of Technology, Śniadeckich 2, 75-453 Koszalin, Poland; andrzej.jacko@gmail.com

**Keywords:** fractional calculus, Caputo derivative, generalized Fourier law, fractional thermoelasticity, stress intensity factor, Laplace transform, Hankel transform, Fourier cosine transform, Mittag-Leffler function

## Abstract

In a real solid there are different types of defects. During sudden cooling, near cracks, there can appear high thermal stresses. In this paper, the time-fractional heat conduction equation is studied in an infinite space with an external circular crack with the interior radius *R* in the case of axial symmetry. The surfaces of a crack are exposed to the constant heat flux loading in a circular ring R<r<ρ. The stress intensity factor is calculated as a function of the order of time-derivative, time, and the size of a circular ring and is presented graphically.

## 1. Introduction

A real solid, as a rule, contains a large number of different type defects: point defects, dislocations, disclinations, slits, inclusions, holes, and cracks. Physical processes occurring in solids depend significantly on the presence of such defects. An external circular crack in an infinite solid under mechanical loading was investigated in [1,2,3,4,5,6,7,8,9,10,11,12,13]. During sudden cooling of a solid, there can arise very high thermal stresses near cracks. For brittle materials, thermal shock is an important fracture mechanism. Starting from the pioneering papers [14,15], the study of thermoelastic problems for solids with cracks has attracted considerable attention from researchers. In the framework of classical thermoelasticity, an external circular crack was considered in [16,17,18,19,20,21,22,23].

The classical thermoelasticity is based on the conventional Fourier law for the heat flux vector and the standard parabolic heat conduction equation for temperature. Many theoretical and experimental studies on heat conduction show that in solids with a complex internal structure, in particular with different types of defects, the Fourier law and the parabolic heat conduction equation should be extended to more general relationships. Different generalizations of the Fourier law and heat conduction equation have been studied intensively in the literature (see [24,25,26,27,28,29,30,31] and references therein). The constitutive equations with memory have considerable promise in this area. It should be emphasized that the concept of memory has found wide use in physics, mechanics, economics, and other fields [32,33,34,35,36,37,38,39,40,41,42]. The generalized Fourier law for the heat flux vector q involving memory effects can be written as

(1) $$q\left(x,t\right)=-k\int_{0}^{t}K\left(t-\tau \right)\;\mathrm{grad}\;T\left(x,\tau \right)\;d\tau$$

and leads to the corresponding heat conduction equation with memory,

(2) $$\frac{\partial T(x,t)}{\partial t}=a\int_{0}^{t}K\left(t-\tau \right)\;\Delta T\left(x,\tau \right)\;d\tau ,$$

where *k* and *a* can be treated as the generalized thermal conductivity and generalized thermal diffusivity coefficient, respectively.

The suitable selection of the memory kernel K(t−τ) allows us to obtain different generalized theories of heat conduction and associated generalized thermoelasticity theories. In the case of “full memory” [26,43], there is no fading of memory, the kernel K(t−τ) is constant, and Equation (2) turns into the wave equation which leads to thermoelasticity without energy dissipation of Green and Naghdi [43]. The exponential memory kernel describes “short-tail memory” and results in the telegraph equation for temperature [24] and generalized thermoelasticity of Lord and Shulman [44] and Green and Lindsay [45].

The theory of integrals and derivatives of fractional order [46,47,48] makes wide use in various areas of science [35,36,39,40,41,42,49,50,51,52,53,54]. The generalization of the Fourier law with the power “long-tail memory” kernel K(t−τ) [39,55,56,57]

(3) $$q\left(x,t\right)=-\frac{k}{\Gamma (\alpha )}\frac{\partial }{\partial t}\int_{0}^{t}{\left(t-\tau \right)}^{\alpha -1}\;\mathrm{grad}\;T\left(x,\tau \right)\;d\tau ,\;0<\alpha \leq 1,$$

(4) $$q\left(x,t\right)=-\frac{k}{\Gamma (\alpha -1)}\int_{0}^{t}{\left(t-\tau \right)}^{\alpha -2}\;\mathrm{grad}\;T\left(x,\tau \right)\;d\tau ,\;1<\alpha \leq 2,$$

can be expressed in terms of derivatives and integrals of the fractional order

(5) $$q\left(x,t\right)=-kD_{RL}^{1-\alpha }\mathrm{grad}\;T\left(x,t\right),\;0<\alpha \leq 1,$$

(6) $$q\left(x,t\right)=-kI^{\alpha -1}\mathrm{grad}\;T\left(x,t\right),\;1<\alpha \leq 2,$$

and gives rise to the time-fractional heat conduction equation

(7) $$\frac{\partial^{\alpha }T\left(x,t\right)}{\partial t^{\alpha }}=a\;\Delta T\left(x,t\right),\;0<\alpha \leq 2,$$

where [46,47,48]

(8) $$I^{\alpha }f\left(t\right)=\frac{1}{\Gamma (\alpha )}\int_{0}^{t}{\left(t-\tau \right)}^{\alpha -1}f\left(\tau \right)\;d\tau ,\;\alpha >0$$

is the Riemann–Liouville fractional integral,

(9) $$D_{RL}^{\alpha }f\left(t\right)=\frac{d^{n}}{dt^{n}}\left[\frac{1}{\Gamma (n-\alpha )}\int_{0}^{t}{\left(t-\tau \right)}^{n-\alpha -1}f\left(\tau \right)\;d\tau \right],\;n-1<\alpha <n$$

denotes the Riemann-Liouville fractional derivative,

(10) $$\frac{d^{\alpha }f\left(t\right)}{dt^{\alpha }}\equiv D_{C}^{\alpha }f\left(t\right)=\frac{1}{\Gamma (n-\alpha )}\int_{0}^{t}{\left(t-\tau \right)}^{n-\alpha -1}\frac{d^{n}f\left(\tau \right)}{d\tau^{n}}\;d\tau ,\;n-1<\alpha <n$$

is the Caputo fractional derivative. Here, Γ(α) is the gamma function.

It should be emphasized that in the constitutive equations for the heat flux (3) and (4), the generalized thermal conductivity *k* has the physical dimension

(11) $$\left[k\right]=\frac{J}{m\cdot s^{\alpha }\cdot K},$$

whereas the generalized thermal diffusivity *a* in the time-fractional heat conduction Equation (7) has the physical dimension

(12) $$\left[a\right]=\frac{m^{2}}{s^{\alpha }}.$$

This will be important in the following when introducing dimensionless quantities (see Equation (35)).

Theory of thermoelasticity associated with the fractional heat conduction Equation (7) was proposed in [55] (see also the review articles [58,59], and the book [39] which sums up investigations in the field of fractional thermoelasticity). The “middle-tail memory” kernel K(t−τ) in the constitutive Equation (1) is expressed in terms of the Mittag-Leffler functions being the generalization of the exponential function. In this case, we arrive at the time-fractional telegraph equations and the associated theories of thermal stresses [60,61].

Cracks in the framework of generalized theories of thermal stresses were studied in [62,63,64,65,66,67]. In the framework of fractional thermoelasticity, line cracks in a plane [68,69,70] and a penny-shaped crack [71] were considered. In the present paper, we expand the previous studies [68,69,70,71] on the case of an external circular crack with the interior radius *R* in an infinite solid under the prescribed heat flux at its surfaces. The surfaces of a crack are exposed to the constant heat flux loading in a circular ring R<r<ρ. The stress intensity factor is calculated as a function of the order of time-derivative, time, and the size of a circular ring and is presented graphically.

## 2. Formulation of the Problem

We consider a space weakened by an external circular crack with the interior radius *R* placed in the plane z=0. The axisymmetric time-fractional heat conduction Equation (7) in the cylindrical coordinates

(13) $$\begin{array}{c} \begin{array}{cc} \frac{\partial^{\alpha }T\left(r,z,t\right)}{\partial t^{\alpha }} & =a\left(\frac{\partial^{2}T\left(r,z,t\right)}{\partial r^{2}}+\frac{1}{r}\;\frac{\partial T(r,z,t)}{\partial r}+\frac{\partial^{2}T\left(r,z,t\right)}{\partial z^{2}}\right)\\ 0\leq r<\infty , & \;-\infty <z<\infty ,\;0<t<\infty ,\;0<\alpha \leq 2, \end{array} \end{array}$$

is studied under zero initial conditions

(14) $$\begin{array}{cc} t=0: & \;T(r,z,t)=0,\;0<\alpha \leq 2, \end{array}$$

(15) $$\begin{array}{cc} t=0: & \;\frac{\partial T(r,z,t)}{\partial t}=0,\;1<\alpha \leq 2, \end{array}$$

and under the prescribed heat flux at the crack surfaces

(16) z=0+:−kDRL1−α∂T(r,z,t)∂z=q(r,t),R<r<∞,0<α≤1,

(17) z=0+:−kIα−1∂T(r,z,t)∂z=q(r,t),R<r<∞,1<α≤2,

(18) $$\begin{array}{cc} z=0^{-}: & \;-kD_{RL}^{1-\alpha }\;\frac{\partial T(r,z,t)}{\partial z}=q\left(r,t\right),\;\;\;R<r<\infty ,\;0<\alpha \leq 1, \end{array}$$

(19) $$\begin{array}{cc} z=0^{-}: & \;-kI^{\alpha -1}\;\frac{\partial T(r,z,t)}{\partial z}=q\left(r,t\right),\;\;\;\;R<r<\infty ,\;1<\alpha \leq 2. \end{array}$$

In the subsequent text, we will consider the constant heat flux $q_{0}$ acting in the local domain R<r<ρ at the crack surface.

Owing to the geometrical symmetry of the equations with respect to the plane z=0, we can simplify the problem. Hence, the time-fractional heat conduction equation is investigated in the upper half-space z>0

(20) $$\begin{array}{c} \begin{array}{cc} \frac{\partial^{\alpha }T\left(r,z,t\right)}{\partial t^{\alpha }} & =a\left(\frac{\partial^{2}T\left(r,z,t\right)}{\partial r^{2}}+\frac{1}{r}\;\frac{\partial T(r,z,t)}{\partial r}+\frac{\partial^{2}T\left(r,z,t\right)}{\partial z^{2}}\right),\\ 0\leq r<\infty , & \;0<z<\infty ,\;0<t<\infty ,\;0<\alpha \leq 2, \end{array} \end{array}$$

under zero initial conditions

(21) $$\begin{array}{cc} t=0: & \;T(r,z,t)=0,\;0<\alpha \leq 2, \end{array}$$

(22) $$\begin{array}{cc} t=0: & \;\frac{\partial T(r,z,t)}{\partial t}=0,\;1<\alpha \leq 2, \end{array}$$

and under the boundary conditions

(23) $$\begin{array}{cc} z=0: & \;kD_{RL}^{1-\alpha }\;\frac{\partial T(r,z,t)}{\partial z}=\left\{\begin{array}{cc} \;q_{0}, & \;R<r<\rho ,\\ \;0, & \;0<r<R,\;\;\rho <r<\infty , \end{array}\right.\;0<\alpha \leq 1, \end{array}$$

(24) $$\begin{array}{cc} z=0: & \;\;\;kI^{\alpha -1}\;\frac{\partial T(r,z,t)}{\partial z}=\left\{\begin{array}{cc} \;q_{0}, & \;R<r<\rho ,\\ \;0, & \;0<r<R,\;\;\rho <r<\infty , \end{array}\right.\;1<\alpha \leq 2. \end{array}$$

The zero conditions at infinity are also imposed:

(25) $$\lim_{r\to \infty }T\left(r,z,t\right)=0,\;\lim_{z\to \infty }T\left(r,z,t\right)=0.$$

In what follows, the Laplace transform with respect to time *t* will be marked by an asterisk, the Hankel transform of the order zero with respect to the radial coordinate *r* will be specified by a hat, and the Fourier cosine transform with respect to the coordinate *z* will be denoted by a tilde (*s*, ξ, and η are the Laplace, Hankel, and Fourier transform variables, respectively).

The Laplace transform rules for the fractional integrals and derivatives have the following form [46,47,48]:

(26) $$L\left\{I^{\alpha }f\left(t\right)\right\}\left(s\right)=\frac{1}{s^{\alpha }}\;f^{*}\left(s\right),$$

(27) $$L\left\{D_{RL}^{\alpha }f\left(t\right)\right\}\left(s\right)=s^{\alpha }f^{*}\left(s\right)-\sum_{k=0}^{n-1}D^{k}I^{n-\alpha }f\left(0^{+}\right)s^{n-1-k},\;n-1<\alpha <n,$$

(28) $$L\left\{\frac{d^{\alpha }f\left(t\right)}{dt^{\alpha }}\right\}\left(s\right)=s^{\alpha }f^{*}\left(s\right)-\sum_{k=0}^{n-1}f^{\left(k\right)}\left(0^{+}\right)s^{\alpha -1-k},\;n-1<\alpha <n.$$

## 3. The Temperature Field

Assuming zero initial conditions for the heat flux, the integral transforms technique gives

(29) $$\hat{\;\tilde{T}}{}^{*}\left(\xi ,\eta ,s\right)=-\frac{aq_{0}}{k\xi }\left[\rho J_{1}\left(\rho \xi \right)-RJ_{1}\left(R\xi \right)\right]\;\frac{s^{\alpha -2}}{s^{\alpha }+a\left(\xi^{2}+\eta^{2}\right)}.$$

Here and in the subsequent text, $J_{n}\left(r\right)$ denotes the Bessel function of the first kind of the order *n*, and the following formula for the Fourier cosine transform of the second derivative

(30) $$F\left\{\frac{d^{2}f\left(z\right)}{dz^{2}}\right\}=-\eta^{2}\tilde{f}\left(\eta \right)-\frac{df(z)}{dz}{|}_{z=0}$$

has been used.

Taking into account that [47,48]

(31) $$L^{-1}\left\{\frac{s^{\alpha -\beta }}{s^{\alpha }+b}\right\}=t^{\beta -1}\;E_{\alpha ,\beta }\left(-bt^{\alpha }\right),$$

where $E_{\alpha ,\beta }\left(z\right)$ is the Mittag-Leffler function in two parameters α and β

(32) $$E_{\alpha ,\beta }\left(z\right)=\sum_{k=0}^{\infty }\frac{z^{k}}{\Gamma (\alpha k+\beta )},\;\alpha >0,\;\;\beta >0,\;\;z\in C,$$

from Equation (29), we obtain

(33) $$\hat{\;\tilde{T}}\left(\xi ,\eta ,t\right)=-\frac{aq_{0}t}{k\xi }\left[\rho J_{1}\left(\rho \xi \right)-RJ_{1}\left(R\xi \right)\right]\;E_{\alpha ,2}\left[-a\left(\xi^{2}+\eta^{2}\right)t^{\alpha }\right].$$

Inverting the Hankel and Fourier transforms allows us to obtain the desired solution of the problem (20)–(25)

(34) $$T\left(r,z,t\right)=-\frac{2aq_{0}t}{\pi k}\int_{0}^{\infty }\int_{0}^{\infty }E_{\alpha ,2}\left[-a\left(\xi^{2}+\eta^{2}\right)t^{\alpha }\right]\left[\rho J_{1}\left(\rho \xi \right)-RJ_{1}\left(R\xi \right)\right]\;J_{0}\left(r\xi \right)\cos\left(z\eta \right)d\xi \;d\eta .$$

The Mittag-Leffler function $E_{1,2}\left(-x\right)$ is expressed as [47,53]

(35) $$E_{1,2}\left(-x\right)=\frac{1-e^{-x}}{x},$$

and after evaluation of the necessary integrals (see Equations (A1) and (A2) in Appendix A), from Equation (34), we obtain the particular case of the solution for the standard parabolic heat conduction equation (α=1):

(36) $$\begin{array}{c} \begin{array}{cc} T(r,z,t) & =-\frac{q_{0}}{k}\int_{0}^{\infty }\left\{e^{-z\xi }-\frac{1}{2}\left[e^{-z\xi }\;\mathrm{erfc}\left(\sqrt{at}\xi -\frac{z}{2\sqrt{at}}\right)+e^{z\xi }\;\mathrm{erfc}\left(\sqrt{at}\xi +\frac{z}{2\sqrt{at}}\right)\right]\right\}\\ \; & \times \left[\rho J_{1}\left(\rho \xi \right)-RJ_{1}\left(R\xi \right)\right]\;\frac{J_{0}\left(r\xi \right)}{\xi }\;d\xi . \end{array} \end{array}$$

**Figure 1:**
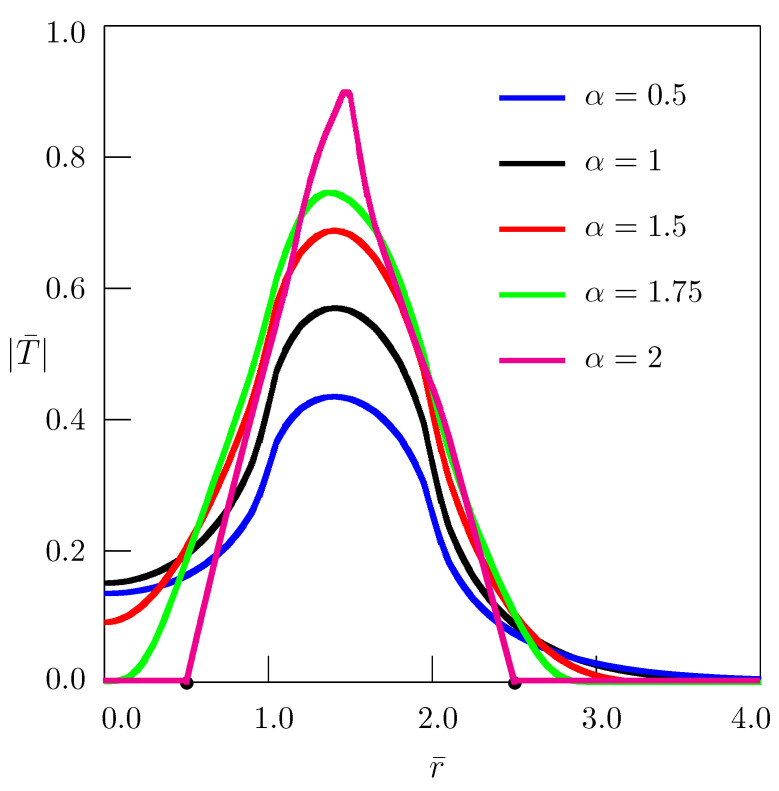
Dependence of temperature on the radial coordinate *r* for z=0 ($\bar{\rho }=2$, $\bar{t}=0.5$).

**Figure 2:**
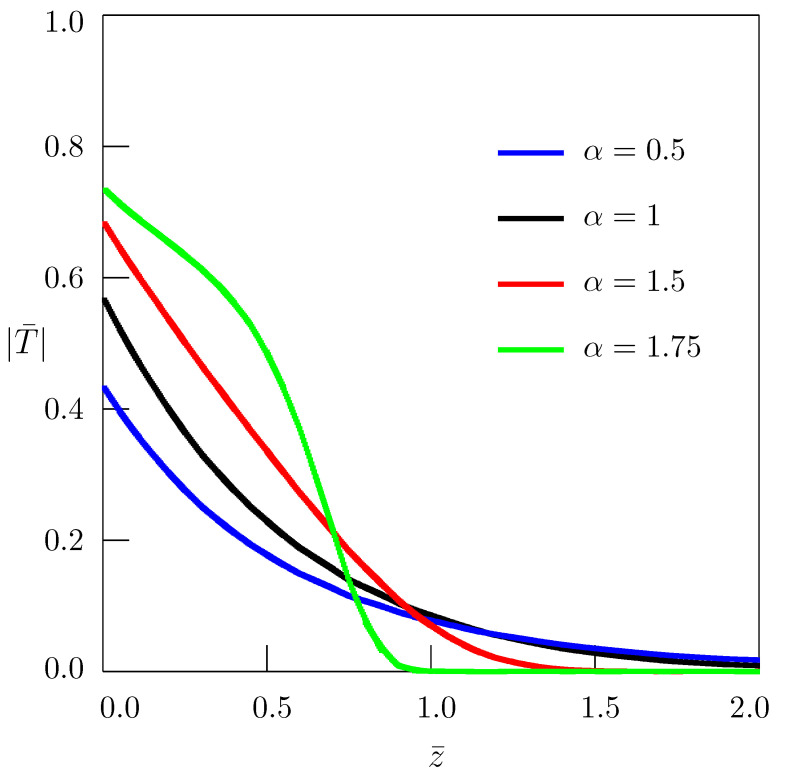
Dependence of temperature on the spatial coordinate *z* for $\bar{r}=1.5$ ($\bar{\rho }=2$, $\bar{t}=0.5$).

Figure 1 shows the dependence of temperature on the radial coordinate *r* for z=0; the curves presented in Figure 2 describe the dependence of temperature on the spatial coordinate *z*. In numerical calculations, the following nondimensional quantities

(37) $$\bar{r}=\frac{r}{R},\;\bar{\rho }=\frac{\rho }{R},\;\bar{z}=\frac{z}{R},\;\bar{\xi }=R\xi ,\;\bar{\eta }=R\eta ,\;\bar{t}=\frac{a^{1/\alpha }}{R^{2/\alpha }}\;t,\;\bar{T}=\frac{kR^{1-2/\alpha }}{q_{0}a^{1-1/\alpha }}\;T$$

have been used (see also Equations (11) and (12)). In the case of the standard heat conduction equation (α=1), the dimensionless time $\bar{t}$ reduces to the Fourier number Fo.

To simplify numerical calculations, it is convenient to pass to the polar coordinate system in the $\left(\bar{\xi },\bar{\eta }\right)$-plane:

(38) $$\bar{\xi }=\zeta \;\cos\theta ,\;\bar{\eta }=\zeta \;\sin\theta .$$

Equation (34), in terms of nondimensional quantities, takes the form

(39) $$\begin{array}{c} \begin{array}{cc} \bar{T}\left(\bar{r},\bar{z},\bar{t}\right) & =-\frac{2\bar{t}}{\pi }\int_{0}^{\infty }\zeta \;E_{\alpha ,2}\left(-\zeta^{2}{\bar{t}}^{\;\alpha }\right)\;d\zeta \\ \; & \times \;\int_{0}^{\pi /2}\left[\bar{\rho }J_{1}\left(\bar{\rho }\zeta \;\cos\theta \right)-J_{1}\left(\zeta \;\cos\theta \right)\right]\;J_{0}\left(\bar{r}\zeta \;\cos\theta \right)\cos\left(\bar{z}\zeta \;\sin\theta \right)d\theta . \end{array} \end{array}$$

## 4. The Stress Intensity Factor

The theory of thermoelasticity associated with the fractional heat conduction Equation (7) was proposed in [55]. The system of basic equations of this theory consists of the equilibrium equation in terms of displacements

(40) $$\mu \Delta u+\left(\lambda +\mu \right)\mathrm{grad}\;\mathrm{div}u=\beta_{T}K_{T}\;\mathrm{grad}\;T,$$

the stress–strain–temperature constitutive equation

(41) $$\sigma =2\mu e+\left(\lambda \;\mathrm{tr}\;e-\beta_{T}K_{T}T\right)I,$$

the geometrical relations

(42) $$e=\frac{1}{2}\left(\nabla u+u\nabla \right),$$

and the time-fractional heat conduction equation

(43) $$\frac{\partial^{\alpha }T}{\partial t^{\alpha }}=a\Delta T,\;0<\alpha \leq 2.$$

Here, σ is the stress tensor, e denotes the linear strain tensor, u is the displacement vector, *∇* is the gradient operator, *T* is the temperature, λ and μ are Lamé constants, $K_{T}=\lambda +2\mu /3$ is the bulk modulus, the thermal coefficient of volumetric expansion is designated by $\beta_{T}$, I stands for the unit tensor.

As the problem is symmetric with respect to the plane z=0, we consider the upper half-space z≥0 under the following boundary condition: by virtue of the fact that the surfaces of the external crack are free of mechanical loading,

(44) $$\begin{array}{cc} z=0: & \;\sigma_{zz}=0,\;R<r<\infty , \end{array}$$

(45) $$\begin{array}{cc} z=0: & \;\sigma_{rz}=0,\;R<r<\infty , \end{array}$$

whereas the geometrical symmetry requirements demand that

(46) $$\begin{array}{cc} z=0: & \;\;u_{z}=0,\;\;\;0\leq r<R, \end{array}$$

(47) $$\begin{array}{cc} z=0: & \;\;\sigma_{rz}=0,\;0\leq r<R. \end{array}$$

The influence of the temperature field on the stress field can be represented by the displacement potential Φ which is introduced similarly to the classical thermoelasticity [72,73]:

(48) $$u^{\left(1\right)}=\mathrm{grad}\;\Phi ,$$

(49) $$\sigma^{\left(1\right)}=2\mu \left(\nabla \nabla \Phi -I\;\Delta \Phi \right)$$

with

(50) $$\Delta \Phi =mT,\;m=\frac{1+\nu }{1-\nu }\;\frac{\beta_{T}}{3}.$$

Here, ν denotes the Poisson ratio.

In the axisymmetric case in cylindrical coordinates, the displacement potential gives the components of the displacement vector

(51) $$\begin{array}{ccc} u_{r}^{\left(1\right)} & \;\;= & \;\;\frac{\partial \Phi }{\partial r}, \end{array}$$

(52) $$\begin{array}{ccc} u_{z}^{\left(1\right)} & \;\;= & \;\;\frac{\partial \Phi }{\partial z}, \end{array}$$

and the components of the stress tensor

(53) $$\begin{array}{ccc} \sigma_{rr}^{\left(1\right)} & \;\;= & \;\;2\mu \left[\frac{\partial^{2}\Phi }{\partial r^{2}}-\Delta \Phi \right], \end{array}$$

(54) $$\begin{array}{ccc} \sigma_{\theta \theta }^{\left(1\right)} & \;\;= & \;\;2\mu \left[\frac{1}{r}\;\frac{\partial \Phi }{\partial r}-\Delta \Phi \right], \end{array}$$

(55) $$\begin{array}{ccc} \sigma_{zz}^{\left(1\right)} & \;\;= & \;\;2\mu \left[\frac{\partial^{2}\Phi }{\partial z^{2}}-\Delta \Phi \right], \end{array}$$

(56) $$\begin{array}{ccc} \sigma_{rz}^{\left(1\right)} & \;\;= & \;\;2\mu \;\frac{\partial^{2}\Phi }{\partial r\partial z}\;, \end{array}$$

where

(57) $$\frac{\partial^{2}\Phi \left(r,z,t\right)}{\partial r^{2}}+\frac{1}{r}\;\frac{\partial \Phi \left(r,z,t\right)}{\partial r}+\frac{\partial^{2}\Phi \left(r,z,t\right)}{\partial z^{2}}=mT\left(r,z,t\right).$$

Assuming that $\frac{\partial \Phi \left(r,z,t\right)}{\partial z}{|}_{z=0}=0$, from Equations (33) and (57), we obtain the expression for the displacement potential in the transform domain

(58) $$\begin{array}{c} \begin{array}{cc} \hat{\tilde{\Phi }}\left(\xi ,\eta ,t\right)\; & =-\frac{m}{\xi^{2}+\eta^{2}}\;\hat{\;\tilde{T}}\left(\xi ,\eta ,t\right)\\ \; & =\;\frac{maq_{0}t}{k\xi }\;E_{\alpha ,2}\left[-a\left(\xi^{2}+\eta^{2}\right)t^{\alpha }\right].\left[\rho J_{1}\left(\rho \xi \right)-RJ_{1}\left(R\xi \right)\right]\;\frac{1}{\xi^{2}+\eta^{2}}\;, \end{array} \end{array}$$

and after inverting the Hankel and Fourier transforms, we obtain

(59) $$\begin{array}{c} \begin{array}{cc} \Phi (r,z,t)\; & =\frac{2maq_{0}t}{\pi k}\int_{0}^{\infty }\int_{0}^{\infty }\frac{1}{\xi^{2}+\eta^{2}}\;E_{\alpha ,2}\left[-a\left(\xi^{2}+\eta^{2}\right)t^{\alpha }\right]\\ \; & \times \;\left[\rho J_{1}\left(\rho \xi \right)-RJ_{1}\left(R\xi \right)\right]J_{0}\left(r\xi \right)\cos\left(z\eta \right)d\xi \;d\eta . \end{array} \end{array}$$

In what follows, we restrict ourselves to calculation of the stress intensity factor which is the most important characteristic of brittle fracture. For the axisymmetric external crack problem in cylindrical coordinates, the stress intensity factor $K_{I}$ describes the singularity of the total stress component $\sigma_{zz}$ at the crack tip [19]

(60) $$K_{I}=\lim_{r\to R^{-}}\sqrt{2\pi (R-r)}\;\;\sigma_{zz}{|}_{z=0}$$

and in terms of the stress component, $\sigma_{zz}^{\left(1\right)}$ is expressed as

(61) $$K_{I}\left(t\right)=\frac{2}{\sqrt{\pi R}}\;\int_{R}^{\infty }\frac{r\;p_{0}\left(r,t\right)}{\sqrt{r^{2}-R^{2}}}\;dr,$$

where

(62) $$p_{0}\left(r,t\right)=\sigma_{zz}^{\left(1\right)}\left(r,0,t\right),\;R<r<\infty .$$

From Equations (55) and (59), it is inferred that

(63) $$\begin{array}{c} \begin{array}{cc} \sigma_{zz}^{\left(1\right)}\left(r,z,t\right)\; & =\frac{4\mu maq_{0}t}{\pi k}\int_{0}^{\infty }\int_{0}^{\infty }\frac{\xi^{2}}{\xi^{2}+\eta^{2}}\;E_{\alpha ,2}\left[-a\left(\xi^{2}+\eta^{2}\right)t^{\alpha }\right]\\ \; & \times \;\left[\rho J_{1}\left(\rho \xi \right)-RJ_{1}\left(R\xi \right)\right]J_{0}\left(r\xi \right)\cos\left(z\eta \right)d\xi \;d\eta \end{array} \end{array}$$

and

(64) $$\begin{array}{c} \begin{array}{cc} K_{I}\left(t\right)\; & =\frac{8\mu maq_{0}t}{k\sqrt{R}\pi^{3/2}}\int_{0}^{\infty }\int_{0}^{\infty }\frac{\xi^{2}}{\xi^{2}+\eta^{2}}\;E_{\alpha ,2}\left[-a\left(\xi^{2}+\eta^{2}\right)t^{\alpha }\right]\\ \; & \times \;\left[\rho J_{1}\left(\rho \xi \right)-RJ_{1}\left(R\xi \right)\right]\;d\xi \;d\eta \int_{R}^{\infty }\frac{r\;J_{0}\left(r\xi \right)}{\sqrt{r^{2}-R^{2}}}\;dr. \end{array} \end{array}$$

Taking into account the integral (A3) from the Appendix A, we arrive at

(65) $$\begin{array}{c} \begin{array}{cc} K_{I}\left(t\right)\; & =\frac{8\mu maq_{0}t}{k\sqrt{R}\pi^{3/2}}\int_{0}^{\infty }\int_{0}^{\infty }\frac{\xi }{\xi^{2}+\eta^{2}}\;E_{\alpha ,2}\left[-a\left(\xi^{2}+\eta^{2}\right)t^{\alpha }\right]\\ \; & \times \;\left[\rho J_{1}\left(\rho \xi \right)-RJ_{1}\left(R\xi \right)\right]\;\cos\left(R\xi \right)\;d\xi \;d\eta \end{array} \end{array}$$

For α=1, using integral (A4) from the Appendix A, we obtain the particular case of Equation (65) corresponding to the classical thermoelasticity:

(66) $$\begin{array}{c} \begin{array}{cc} K_{I}\left(t\right) & =\frac{2\mu mq_{0}}{k\sqrt{\pi R}}\int_{0}^{\infty }\left[\mathrm{erf}\left(\sqrt{at}\xi \right)+2at\xi^{2}\mathrm{erfc}\left(\sqrt{at}\xi \right)-\frac{2\sqrt{at}\xi }{\sqrt{\pi }}\;\exp\left(-at\xi^{2}\right)\right]\\ \; & \times \;\left[\rho J_{1}\left(\rho \xi \right)-RJ_{1}\left(R\xi \right)\right]\cos\left(R\xi \right)\frac{1}{\xi^{2}}\;d\xi . \end{array} \end{array}$$

After passing to the polar coordinate system in the $\left(\bar{\xi },\bar{\eta }\right)$-plane, Equation (65) is rewritten in nondimensional form as

(67) $$\begin{array}{c} \begin{array}{cc} {\bar{K}}_{I}\left(\bar{t}\right)\; & =\frac{4\bar{t}}{\pi^{3/2}}\int_{0}^{\infty }E_{\alpha ,2}\left(-\zeta^{2}{\bar{t}}^{\;\alpha }\right)d\zeta \\ \; & \times \;\int_{0}^{\pi /2}\left[\bar{\rho }J_{1}\left(\bar{\rho }\zeta \cos\theta \right)-J_{1}\left(\zeta \cos\theta \right)\right]\;\cos\left(\zeta \cos\theta \right)\cos\theta \;d\theta . \end{array} \end{array}$$

**Figure 3:**
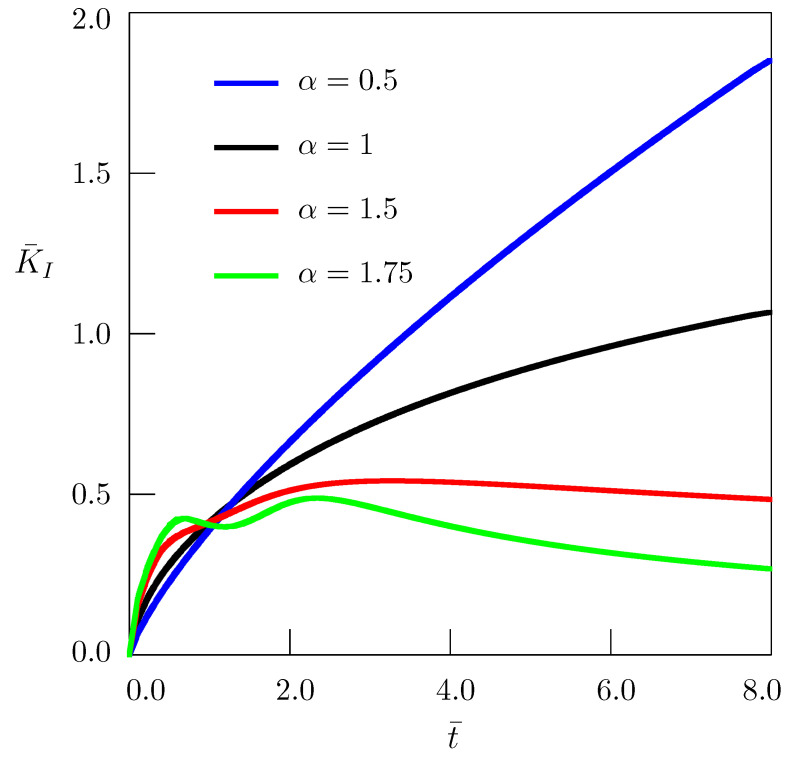
Dependence of the stress intensity factor on time for $\bar{\rho }=2$.

**Figure 4:**
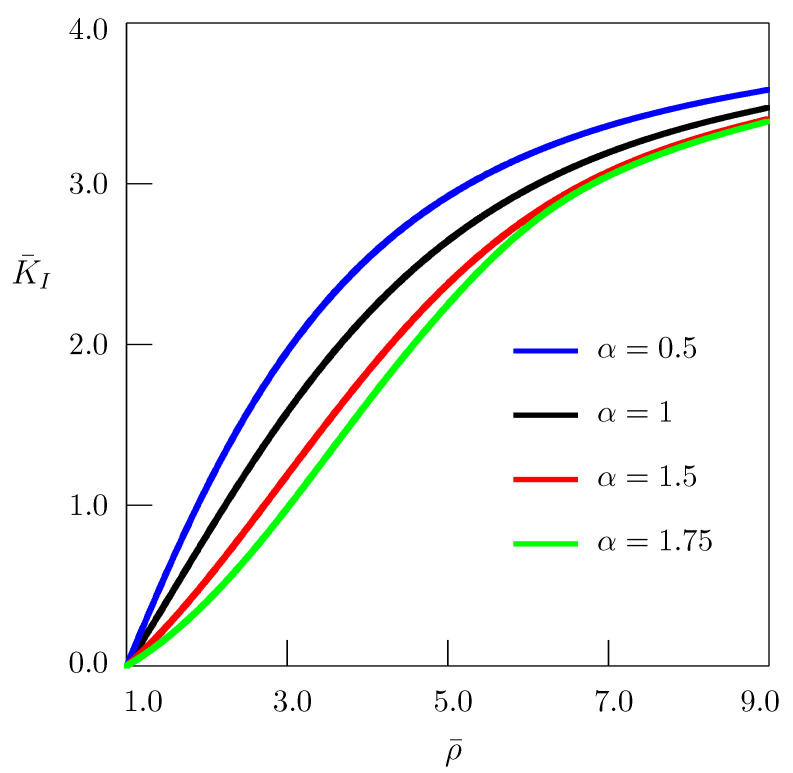
Dependence of the stress intensity factor on the parameter $\bar{\rho }$ for $\bar{t}=4$.

The nondimensional stress factor

(68) $${\bar{K}}_{I}=\frac{kR^{1/2-2/\alpha }}{2\mu mq_{0}a^{1-1/\alpha }}\;K_{I}$$

is presented in Figure 3 as a function of nondimensional time $\bar{t}$, and in Figure 4 as a function of the nondimensional parameter $\bar{\rho }$.

To calculate the total stress field satisfying the boundary conditions (44)–(47), the biharmonic Love function can be used. The interested reader is referred to [73] for general equations and to the paper [71], where this approach was used for a penny-shaped crack.

## 5. Concluding Remarks

We have solved the time-fractional heat conduction equation in an infinite solid with an external circular crack with the interior radius *R* under constant axisymmetric heat flux acting in a circular domain R<r<ρ. The heat flux vector is expressed in terms of the Riemann–Liouville fractional integrals and derivatives of temperature gradient which results in the time-fractional heat conduction equation with the Caputo fractional derivative.

It is worth noting that equations containing the Caputo derivative can be recast to the Riemann–Liouville version (and vice versa) according to the following equation [48,74]:

(69) $$D_{RL}^{\alpha }f\left(t\right)=D_{C}^{\alpha }f\left(t\right)+\sum_{k=0}^{n-1}\frac{t^{k-\alpha }}{\Gamma (k-\alpha +1)}\;f^{\left(k\right)}\left(0^{+}\right),\;n-1<\alpha <n.$$

The temperature field and the stress intensity factor are given as integrals with integrands being the Mittag-Leffler functions in two parameters. To evaluate the Mittag-Leffler function $E_{\alpha ,2}$, we have used the algorithm in [74]. The Mittag-Leffler function $E_{1/2,2}\left(-x\right)$ is evaluated according to the relation [53]

(70) $$E_{1/2,2}\left(-x\right)=\frac{1}{x^{2}}\left[\frac{2x}{\sqrt{\pi }}+e^{x^{2}}\;\mathrm{erfc}\left(x\right)-1\right].$$

The solution of the classical thermoelasticity problem for the external circular crack is obtained as a particular case when the order of time-derivative α=1.

The solutions of various initial-boundary value problems for the time-fractional heat conduction Equation (7) are expressed in terms of the Mittag-Leffler functions $E_{\alpha ,\beta }\left(-t^{\alpha }\right)$ (see Equation (31)). From the analysis of integral representation of $E_{\alpha ,\beta }\left(-t^{\alpha }\right)$ [46,53,74], it follows that for 1<α<3 there appears an expression

(71) $$\exp\left[t\cos\left(\frac{\pi }{\alpha }\right)\right]\cos\left[t\sin\left(\frac{\pi }{\alpha }\right)\right].$$

For the values 1<α<2, we have damped oscillations, but for 2<α<3 there arise amplified oscillations. For this reason, the time-fractional heat conduction Equation (7) is considered for the order of fractional derivatives 0<α≤2.

The time-fractional heat conduction equation for 1≤α≤2 interpolates between the diffusion equation (α=1) and the wave equation (α=2) that behave quite differently with respect to their response to a disturbance. The standard heat conduction equation describes a process where a disturbance spreads infinitely fast, whereas in the case of the wave equation the propagation speed of the disturbance is constant. In Figure 1, the bullet points on the *r*-axis indicate the wave fronts of the solution to the wave equation (α=2) at r=R−ct and r=ρ+ct, where the coefficient *a* can be interpreted as $a=c^{2}$ with *c* being the velocity of wave propagation. In terms of dimensionless quantities, the wave fronts arise at $\bar{r}=1-\bar{t}$ and $\bar{r}=\bar{\rho }+\bar{t}$.

It is seen from Figure 3 that the stress intensity factor depends significantly on the order of fractional derivatives which corresponds to the power “long-tail memory” kernel K(t−τ). For large values of time, when 0<α<1 the stress intensity factor is greater than that for the standard heat conduction (α=1), whereas for 1<α≤2 the stress intensity factor is smaller than that for α=1. The slow heat diffusion (0<α<1) increases the stress intensity factor, the fast heat diffusion (1<α≤2) decreases it. The influence of other memory types, in particular “middle-tail memory”resulting in the time-fractional telegraph equation, will be considered in future research.

## Data Availability

Not applicable.

## Appendix A

Integrals (A1) and (A2) are taken from [75], integral (A3) is borrowed from [76], integral (A4) has been evaluated in this paper.

(A1) $$\int_{0}^{\infty }\frac{1}{x^{2}+c^{2}}\;\cos\left(bx\right)dx=\frac{\pi }{2c}\;e^{-bc},\;b\geq 0,\;\;c>0,$$

(A2) $$\begin{array}{c} \int_{0}^{\infty }\frac{1}{x^{2}+c^{2}}\;e^{-a^{2}x^{2}}\;\cos\left(bx\right)dx=\frac{\pi }{4c}\;e^{a^{2}c^{2}}\left[e^{-bc}\;\mathrm{erfc}\left(ac-\frac{b}{2a}\right)+e^{bc}\;\mathrm{erfc}\left(ac+\frac{b}{2a}\right)\right],\\ \;a\geq 0,\;\;c>0, \end{array}$$

(A3) $$\int_{a}^{\infty }\frac{x}{\sqrt{x^{2}-a^{2}}}\;J_{0}\left(cx\right)dx=\frac{\cos\left(ac\right)}{c},\;a>0,\;\;c>0,$$

(A4) $$\int_{0}^{\infty }\frac{e^{-a^{2}x^{2}}}{{\left(x^{2}+c^{2}\right)}^{2}}\;dx=\frac{\pi }{4c}\left[\left(\frac{1}{c^{2}}-2a^{2}\right)e^{a^{2}c^{2}}\;\mathrm{erfc}\left(ac\right)+\frac{2a}{\sqrt{\pi }c}\right],\;a\geq 0,\;\;c>0.$$

Here, $\mathrm{erfc}\left(x\right)=1-\mathrm{erf}\left(x\right)$ is the complementary error function.
